# Increased perioperative mortality for femoral neck fractures in patients with coronavirus disease 2019 (COVID-19): experience from the United Kingdom during the first wave of the pandemic

**DOI:** 10.1186/s13037-020-00279-x

**Published:** 2021-01-10

**Authors:** Esther Victoria Wright, Omar Musbahi, Abhinav Singh, Naresh Somashekar, Christopher P. Huber, Anatole Vilhelm Wiik

**Affiliations:** 1 The Hillingdon Hospitals NHS Foundation Trust, Uxbridge, UK; 2 Imperial College London, London, UK; 3 West Middlesex University Hospital, Chelsea and Westminster NHS Foundation Trust, Middlesex, UK; 4 Northwick Park Hospital NHS Foundation Trust, Harrow, UK

**Keywords:** COVID-19, Trauma, Orthopaedics, Neck of femur, Fracture, Orthogeriatrics, Frailty, Patient safety

## Abstract

**Background:**

The coronavirus disease 19 (COVID-19) pandemic has presented modern healthcare with an unprecedented challenge. At the peak of the pandemic, trauma and orthopaedic services at our institutions undertook internal restructuring, diverting resources to frontline medical care. Consequently, we sought to assess the impact on the elderly and comorbid patients presenting with femoral neck fractures, with a particular focus on 30-day mortality, length of stay, multidisciplinary team involvement and departmental structuring.

**Method:**

A retrospective analysis of patients presenting with femoral neck fractures at three separate West London NHS Trusts was undertaken between March 11, 2020, to April 30, 2020. Length of stay, 30-day mortality and adherence to parameters constituting the best care evidence-based practice tariffs were compared between severe acute respiratory syndrome coronavirus 2 (SARS-CoV-2) positive and negative patients. A similar comparison was also conducted between our cohort and the equivalent period in 2018 using data from the National Hip Fracture Database.

**Results:**

A total of 68 patients presenting with femoral neck fractures were identified, mean age 81 (range 38–98), 73% female. There were 10 confirmed/suspected cases of COVID-19 on admission and a further seven confirmed as inpatients. The 30-day mortality within our cohort was 11.76% compared to 6% nationally in 2018 (*p* = 0.045). Orthogeriatric reviews occurred within 72 h in 71% of cases compared to 88% in the equivalent 2018 period. Within the cohort, mean length of stay was 17.13 days (SD 5.6, range 8-27 days) for SARS-CoV-2 positive patients compared to 10 days (SD 8.7, range 1–53 days) for negative patients (*p* < 0.05). Thirty-two patients (47%) required increased packages of care on discharge or rehabilitation.

**Conclusions:**

The increase in 30-day mortality for SARS-CoV-2 positive patients presenting with femoral neck fractures is multifactorial, resulting from a combination of the direct effects of COVID-19 pneumonia as well as changes to the delivery of orthopaedic services. The provision of multidisciplinary care was directly affected by staff redeployment, particularly reorganisation of orthogeriatric services and lack of continuity of ward based clinical care. Our experiences have re-directed efforts towards the management of theatre teams, patient services and staffing, should we be faced with either a resurgence of COVID-19 or a future pandemic.

## Introduction

The severe acute respiratory syndrome coronavirus 2 (SARS-CoV-2), resulting in the coronavirus disease 19 (COVID-19), has spread globally with unprecedented speed and magnitude. Within the first 6 months of 2020, there have been 8,242,999 cases with 445,535 deaths worldwide [1]. COVID-19 has had dramatic effects on healthcare and the National Health Service (NHS), due to the difficulty of ensuring safe patient care whilst combating the co-morbid effects of this unknown virus.

COVID-19 is a highly transmissible disease associated with high mortality in specific patient groups (obese, elderly, immunocompromised). Viral spread is primarily via aerosols of the nasal and respiratory tract, alongside additional methods including direct contact and surface transmission [2, 3]. Studies have shown that the viral load remains equivocal between asymptomatic and symptomatic carriers, making contact tracing problematic [2]. Epidemiological data has also shown that hospital staff are at increased risk of COVID-19 infection, adding to the complexity of orthopaedic service provision [4]. Furthermore, elderly (> 65 years) and comorbid patients; those who are obese, immunosuppressed and have pre-existing respiratory disease, are particularly susceptible [5]. Consequently, within trauma and orthopaedics units, the population presenting with femoral neck fractures who fall into this category, are considered to be highly vulnerable. Typically, these patients have low functional status, are highly comorbid and require greater medical input during hospital admission [6].

Prior to the COVID-19 pandemic, the management of patients with femoral neck fractures was centred around the British Orthopaedic Association endorsed National Institute for Health and Care Excellence (NICE) guidelines [7]. These guidelines were governed through the implementation of a National Hip Fracture Database(NHFD), which forms part of the Falls and Fragility Fractures Audit Programme [8]. The database records a number of key outcome measures including; prompt orthogeriatric assessment, timely surgery, screening for pre and post-operative delirium and rehabilitation and community service involvement [9]. These criteria form the foundations of the best care evidence-based practice tariffs. These guidelines also offer healthcare providers financial incentives for delivering optimal care which is proven to improve neck of femur fracture outcomes [10, 11].

During the pandemic, these tariffs have been intermittently suspended, most likely in anticipation of the diversion and redeployment of services to frontline acute medical care. Consequently, many orthopaedic services underwent internal restructuring, leading to the reduction in both elective and trauma capacity, in order to accommodate additional intensive care facilities [12]. New recommendations for the management of all trauma and orthopaedic patients during the pandemic were published. This guidance included advising surgical planning to permit immediate post-operative weight bearing, and considering non-operative management of fractures where appropriate [13]. The intentions were to promote early rehabilitation and reduce inpatient stays, aiming to limit potential COVID-19 exposure and its associated mortality.

To date, no study has addressed the internal restructuring of trauma and orthopaedic services within the NHS. This retrospective audit analysis specifically addressed the management and outcomes of patients presenting with femoral neck fractures at three district general hospitals at the epicentre of the COVID-19 pandemic in London. The aim was to compare the established mortality of patients presenting with femoral neck fractures during the COVID-19 pandemic to the equivalent period in 2018 using the data from the NHFD. Based upon these results experiences, recommendations are made to improve patient outcomes in this vulnerable population in the event of a second surge of cases or further pandemics.

## Methods

### Study design

A retrospective audit analysis of local trauma units at three separate West London NHS Trusts was undertaken. All patients presenting with femoral neck fractures in the period between March 11, 2020, to April 30, 2020 were identified. This period was in line with the initial lockdown announcement and covered the peak of the first wave of the pandemic.

### Data collection

Inclusion criteria was any patient presenting with a proximal femoral fracture. Distal femoral fractures and periprosthetic femoral fractures were excluded. Baseline demographic data was collected. Routine parameters as indexed by the NHFD were also collected. Other data points included in the analysis included the routine British Orthopaedic Association standards of care for neck of femur fracture: Orthogeriatric review, venous thromboembolism assessment, and 30-day mortality. SARS-CoV-2 status was also assessed. A patient was considered positive if they had a SARS-CoV-2 positive swab result (RT-PCR) or presented with features of suspected COVID-19 disease, including lymphopenia, chest radiograph changes and respiratory symptoms (cough, fever, shortness of breath). Sample collection was carried out using Sigma Virocult® swabs (Medical Wire and Equipment, England) and results were available within 36 h of collection, although this time was variable. This is in keeping with the NHS practice at the time of this study, where routine testing was not mandatory for inpatients all at our sites, although 92.6% of our cohort did undergo COVID-19 RT-PCR testing during the course of their admission. Admission and operative details including time to operation and length of stay (LOS) was also recorded.

### Statistical analysis

Patient demographic data was recorded as descriptive statistics. The patient sample during the sample period (April 2020) was compared to the national average in April 2018 (pre-COVID era). The comparison included age, gender and LOS.

A comparison between the COVID 30-day mortality for all neck of femur patients and the pre-COVID mortality was also done using the 2019 annual NHFD report [14]. Shapiro-Wilk test was used to test for normality. A multivariate logistic regression model with 30-day mortality treated as the outcome variable and COVID status as the dependent variable was undertaken. Postestimation analysis was undertaken using likelihood ratio test to compare each model. A two-sample T test was used to compare the length of stay for SARS-CoV-2 positive patients and negative patients.

The results of best practice performance and criteria was compared to the same period pre-COVID in 2018 using the national audit data for each site. A one-way test of proportions or a one-way samples T test was performed to compare the COVID period parameters with the published pre-COVID period statistics and national audit standards.

STATA™ Software (STATA Corp, USA) was used for statistical analysis of primary data.

### Recommendations

This study presents recommendations based on the experiences of managing patients presenting with femoral neck fractures during the COVID-19 pandemic. All three orthopaedic units’ views and experiences were considered in this article. Given the limited evidence available, our recommendations were based on an internal consensus.

## Results

### Patient demographics

A total of 68 patients presenting with femoral neck fractures were included over the six-week period with a mean age of 81 (range 38–98), 50 (73%) patients were female and 18 (27%) male (Table 1). Only two cases were managed non-operatively. The average time to theatre was 1.2 days (range 0–6 days). There were 10 (15%) confirmed/suspected cases of COVID-19 on presentation to hospital.

**Table 1:** Patient demographics and SARS-CoV-2 status of neck of femur patients seen during the study period at all three hospitals

Number of Patients	68
Age, *mean (SD)*	81.1 (±11.35)
Gender, *n (%)*	
Male	18 (27%)
Female	50 (73%)
Laterality, *n (%)*	
Right	30 (44.1%)
Left	38 (55.9%)
Charlson Comorbidity Index, *n*(%)	
< =4	34 (50%)
> 4	34 (50%)
ASA, *n* (%)	
1	4 (6%)
2	18 (27%)
3	42 (62%)
4	4 (6%)
SARS-CoV-2 Status, *n* (%)	
Positive	17 (25%)
Negative	51 (75%)
SARS-CoV-2 positive on admission	10 (15%)

### Determinants of in-hospital mortality 2020

#### Outcomes for SARS-CoV-2 positive patients

The average time to length of stay, from Emergency Department (ED) presentation to ward discharge, for SARS-CoV-2 positive patients was 17 days. The 30-day mortality was 11.76%. Average post-operative length of stay was 12 days (range 1–53) (Table 2). Thirty-two patients (47%) required greater support at home or a higher level of care on discharge. One-way test of proportions comparing our cohort 30-day mortality (11.76%) to that of the equivalent 2018 period pre-COVID cohort in the NHFD (6%) was significant (*p* = 0.045). Orthogeriatric reviews were conducted in 57 of the 68 admissions; 71% of patients were reviewed within 72 h of presentation. Venous Thromboembolism assessment scores were recorded for all admissions across sites.

**Table 2:** Table highlighting Admission, operative and outcome information

	SARS-CoV-2 negative	SARS-CoV-2 positive
Length of Stay	Mean 10 days (SD 8.7, range 1–53 days)	Mean 17 days (SD 5.6, range 8-27 days)
Operation		
•Hemiarthroplasty	29	5
•Dynamic hip screw	16	6
•Intramedullary nail	4	5
•Total hip arthroplasty	1	0
Non-operative management	1	1
Orthogeriatric assessment during admission	44 (86.3%)	13 (76.5%)
ICU admission	0	0
Admission to theatre time	1 day (SD 0.93)	1.76 days (SD 1.6)
Mechanism of Injury (Fall from < 2)	51	16 (1 assault)
30-day mortality	3	5

**Fig. 1:**
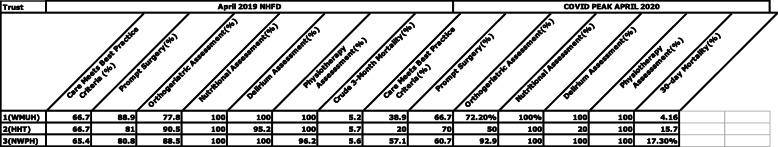
Results of the April 2020 COVID study period across our three sites compared to the equivalent 2019 NHFD

The multivariate regression model showed that of the parameters assessed, only SARS-CoV-2 status was statistically significant and associated with higher in-hospital mortality (95% CI 1.54–61.4). Age, sex and pre-operative haemoglobin had no correlation with in-hospital mortality. The adjusted and unadjusted odds ratio for the model are shown in Table 3. A positive SARS-CoV-2 status was associated with an increased LOS (means difference 6.5 days, *P* < 0.05, 95% CI (1.7–11.2). Figure 1 shows the best practice tariff audit comparison between the three sites pre-COVID and during the COVID-19 period.

**Table 3:** Multivariate Logistic regression model with the In-hospital mortality as dependent variable with the associated crude and adjusted Odds Ratios

In-Hospital Mortality	Crude Odds Ratio	Adjusted Odds Ratio	*P* value	95% Confidence Interval
SARS-CoV-2 Status	6.67	9.58	< 0.05	1.54–61.4
Age	1.04	1.05	0.329	0.95–1.18
ASA Grade 2	1.21	1.46	0.699	0.21–10
Sex (Female)	1.09	0.42	0.784	0.047–3.87
Pre-operative Haemoglobin	0.99	1.0	0.784	0.95–1.07

### Comparison of best practice standards

Fig. 1

## Discussion

### Patient demographics

The demographic of patients presenting with femoral neck fractures during the COVID-19 outbreak was in keeping with the pre-pandemic population (Table 1) [15]. Nationwide, orthopaedic trauma admissions were decreased [16, 17]. Within the studied centres, the number of patients admitted with femoral neck fractures averaged at 3.78 per site per week during the six-week period, a slight decrease compared to 4.71 across 2019 [14]. This may be explained by the effects of the social lockdown measures in place. As such, significant changes in mortality are likely explained by external factors. Apart from one patient, all femoral neck fractures were sustained from a fall from standing height or greater. As identified from General Practitioner records, only 13 patients had a formal diagnosis of dementia, six were known to have osteoporosis and 68% of the cohort had more than three comorbidities at time of presentation. Recent evidence suggests poor post-operative outcomes for patients with reduced pre-fracture baseline functional status, both cognitively and physically [18, 19].

Despite the apparent benefits of sharing theatre resources, considerations such as cross contamination of patients and the subsequent transmission of COVID-19 was an important consideration. Although 10 patients had tested SARS-CoV-2 positive prior to or at admission, a further seven contracted the virus whilst inpatients, representing 25% of the total population. Six of the seven patients testing positive as inpatients received the positive diagnosis post-operatively, meaning that they may have been infected at the time of surgery. At the outset of the pandemic, SARS-CoV-2 real time polymerase chain reaction (RT-PCR) testing took over 24 h to generate results, meaning that emergency procedures were carried out on patients of unknown COVID status. Testing is now achievable in less than 90 min across all sites, although with limited capacity. The timing of RT-PCR testing is crucial to avoidance of false negative results; nasopharyngeal samples are most likely to be positive within two weeks of symptoms onset [20]. Repeated swabs may be considered in symptomatic individuals if the diagnosis is still suspected despite a previous negative swab, furthermore regularly testing patients with prolonged admissions, such as those with femoral neck fractures, may be beneficial [21].

### Determinants of 30-day mortality

Overall 30-day mortality for the COVID-19 study period was increased (11.76%) when compared to the national average in 2019 (6%) [14]. The results suggest this may be explained directly by the effects of COVID-19 pneumonia. Current evidence indicates that approximately half of SARS-CoV-2 positive patients undergoing surgery will experience pulmonary complications, with a statistically significant increase in 30-day mortality for those over 70 [22]. Furthermore, three patients had delays in operative management secondary to complications of COVID pneumonia and a further patient was managed non-operatively as they were deemed too unwell to undergo anaesthesia. This is reflected in our own mortality data, demonstrating that over half of deaths within our population were associated with a positive COVID status. In a retrospective study of 10 SARS-CoV-2 positive patients that underwent hospitalisation for fractures, Mei et al. also reported increased mortality after open reduction and internal fixation [23]. Increased mortality has also been reported by Hall et al. demonstrating a 30 day mortality of 35.5% for SARS-CoV-2 positive patients compared to 8.3% for negative in a study of 317 patients presenting with acute hip fractures [24]. Early surgery is associated with reduced mortality in patients with a fractured neck of femur, as reflected by the best practice tariff of operative management within 36 h of admission [25]. As such, COVID-19 related sequelae may be both cause and effect of delays to theatre.

COVID status also impacted LOS, with a mean LOS of 17.13 (SD 5.6, range 8-27 days) days for positive patients compared to SARS-CoV-2 negative patients (mean LOS 10.64 range 1–53 days) (*p* < 0.05). The cause for this discrepancy is likely multifactorial. Confirmed positive patients required side rooms and barrier nursing, and although physiotherapy was still indicated, this may have limited their post-operative mobility and complicated early mobilisation [26]. Furthermore, unwell patients who did not return to their baseline functional status following admission required input from rehabilitation facilities or increased packages of care, which can delay discharge. Of our cohort, 47% required greater support at home or a higher level of care on discharge. Of the 17 patients testing positive for COVID-19 only 4 were discharged to their admission residence. The number of patients stepped down to residential and rehabilitation facilities, which reportedly cared for high numbers of SARS-CoV-2 positive patients, may further explain the overall increase in mortality for this cohort [27].

### Comparison of best practice audit standards

The COVID updated British Orthopaedic Association guidance, advising operative management to restore weight bearing status as a priority was followed in all eligible cases. All methods of fixation utilised ensured immediate weight bearing potential and half of cases were managed with hemiarthroplasty. The ultimate aim was to reduce LOS and subsequent in-patient exposure to COVID-19 [28]. The NHFD has reported gradually declining average length of stays since March 2020, falling from 15.4 to 14.7 in July, the lowest on record since 2012, suggesting that this practice is being followed nationwide. Of the patients managed non-operatively within our population, one was identified as having a severe COVID infection and as such was deemed too high risk for either spinal or general anaesthesia. A further patient with presumed COVID-19 died pre-operatively of respiratory complications.

The multidisciplinary approach to managing patients with femoral neck fractures is associated with improved functional outcome and reduced mortality [29, 30]. Best practice criteria include a review by an orthogeriatric registrar or consultant within 72 h of admission. Prior to staff redeployment during the pandemic, all three sites had dedicated orthogeriatric services, providing daily patient input. Throughout the redeployment period, review frequency was reduced by over half, with on call medical teams providing advice only when required. During March 2018, best practice criteria were achieved in 88.8% across the three sites compared to 71% in our cohort. This relative delay in expert medical input may also have contributed to the apparent rise in mortality during this time. Furthermore, patient care was severely disrupted during this period, with senior orthopaedic surgeons managing the majority of patients’ ward care on a rolling daily rota. As determined by Van Walraven et al., continuity of care is associated with improved patient outcome and satisfaction post-operatively [31]. Consequently, the restructuring of essential services staffing may have impacted the care for patients with femoral neck fractures.

The NICE and British Orthopaedic Association 2017 quality standards recommend early mobilisation following hip fractures [7]. The role of the physiotherapist and occupational therapist here is crucial. Through early mobilisation, we limit the time spent in hospital and hence reducing the susceptibility of all patients regardless of COVID status [32]. Diligent surgical planning ensured immediate post-operative partial or full weight bearing status was achieved for all patients. Post-operative physiotherapy assessment was achieved in 100% of patients across sites, ensuring both prompt and safe mobilisation.

### Recommendations of femoral neck fracture management and services

All three sites undertook internal orthopaedic team restructuring during the peak of the pandemic. Whilst only essential elective services were retained, emergency (CEPOD) and trauma theatres were amalgamated into one service to accommodate additional intensive care facilities. Despite this change, the mean time to theatre remained an acceptable 1.2 days when average across the three sites. This may be related to the relative decrease in general hospital admissions, reducing competition for theatre time. In addition, respective Royal Colleges guidance advised non-operative management of common pathologies including distal radius fractures and non-complicated acute appendicitis, further reducing the demand on emergency theatre provision [13, 33, 34].

Departmental restructuring across sites ensured senior decision making available around the clock, ensuring review by a registrar at initial presentation of a patient with a femoral neck fracture to ED.

Division of services ensured presence of a senior led on-call team, a separate trauma team and the establishment of virtual fracture clinic facilities. Furthermore, in keeping with social distancing guidance, teams were advised to limit non-essential contact with each other. In line with the global report on safe surgical practice during COVID, NHS trusts provided temporary housing for the acute surgical workforce if required [35].

Our experiences of managing orthopaedic trauma during the peak of the COVID-19 pandemic have enabled the generation of suggestions for service improvement should society be faced with a further surge in cases. Recommendations are provided for the management of theatre services, patient services and staffing (Table 4).

**Table 4:** Key elements summary for improving fractured neck of femur patient outcomes during a pandemic

**Patient Pathway**	
• Patients with femoral neck fractures are a highly vulnerable cohort and should be treated as such	
• In agreement with the worldwide initiative “best practice for surgeons, COVID-19 evidence based review”, all patients should have their temperature checked and a surgical mask provided on admission [35].	
• Patients admitted from the Emergency Department (ED) who are awaiting a test result should be isolated in a separate bay until the SARS-CoV-2 status is concluded and unnecessary patient transfers should be prevented.	
• High index of suspicion is warranted for patients with atypical symptoms due to the high aerosol risk and poor patient outcomes as detailed above [36].	
• SARS-CoV-2 status should be checked on a regular basis for long staying inpatients	
**Theatre Pathway**	
• Adaptation of the WHO checklist is recommended, to include the addition of “COVID status” and “PPE check”	
• Theatres should have negative pressure room ventilation, and aerosol generating tools e.g. oscillating saws should be used only in necessity [37, 38]	
• One dedicated emergency theatre during peak pandemic time to be expanded to a COVID and Clean theatre where possible	
• Surgeons should use familiar metalwork and trauma hardware. A COVID-19 pandemic is not the ideal period to trial new surgical components or techniques and presence of additional personnel in theatre e.g. representatives of manufacturers should be avoided.	
**Staffing**	
• Consultant services where possible to reduce patient waiting time or delays to decision making	
• Minimal personnel in theatre where possible	
• MDT members including physiotherapy, occupational therapy and orthogeriatric and fracture liaison services must prioritise patients with a fractured neck of femur	
• Restructuring the teams into: “Operating Team”, “On-call team” and “Clinic team” and a “Reserve Team”.	

Other studies looking at different aspects of orthopaedic services during the COVID period have made similar recommendations. Zahra et al. have advised pre-screening all spine patients before elective spinal surgery [39]. The early experiences from Singapore orthopaedic service recommendations are to ensure that there is minimal impact on services, day-case procedures were not altered [40]. In the US, Afshin et al. formulated a list of procedures by urgency, and agree with a similar set of consensus to the British Orthopaedic Association guidelines and our own recommendations that neck of femur fracture surgery should be performed within 1–2 days [41]. Similarly, a study by Service et al. have produced a classification system detailing the steps necessary required for high risk SARS-CoV-2 positive patients [42]. Overall, these study results and recommendations add to the available information and published guidelines to inform local orthopaedic trauma units on the management of this vulnerable patient group. The current study findings advocate adherence to best practice standards as closely as possible within the constraints of available pandemic resources.

### Limitations

This was a retrospective audit study and hence there are the standard limits associated with data collection and analysing of observational data. Acknowledged limitations include the lack of mandatory COVID PCR testing initially for inpatients at the time of this study (92.6% of our patients were tested during admission); there is a small risk of false positive data inclusion. It should also be noted that the study only addressed 30-day mortality data; hence it is possible that the overall long-term clinical outcomes of SARS-CoV-2 positive patients may be different.

## Conclusion

The COVID-19 pandemic had an impact on hip fracture outcomes at three London NHS institutions. Pre-COVID standards and best practice metrics were met by only 45.9% of hospitals nationally in April 2020, compared to 58.5% in April 2019. Despite a small reduction in absolute numbers of femoral neck fractures during this period, presentations remained frequent and represented a greater proportion of trauma admissions. Our study showed a higher mortality (11.76%) in this vulnerable patient group when compared to national pre-COVID data (6%). Furthermore, positive SARS-CoV-2 status was associated with a statistically increased 30-day mortality when compared to negative patients within our cohort (*p* = 0.042). Positive SARS-CoV-2 status was also associated with an increased mean LOS of 17.13 (SD 5.6, range 8-27 days) compared to negative patients (mean LOS 10.64 range 1–53 days) (*p* < 0.05).

Despite adequate performance in timing of operative intervention, a number of pitfalls in care were identified including availability of expert medical opinion and timely diagnosis of COVID infection. The recommendations made in this study will aid in re-organisation of trauma care to this vulnerable patient group to improve outcomes in the event of a further pandemic.

## Data Availability

The datasets generated and/or analysed during the current study are available in the National Hip fracture database repository, https://www.nhfd.co.uk/20/hipfractureR.nsf/docs/2019Report Further datasets used and/or analysed during the current study are available from the corresponding author on reasonable request.

## Abbreviations

SARS-CoV-2: Severe acute respiratory syndrome coronavirus 2
COVID-19: Coronavirus disease 2019
NHS: National Health Service
NHFD: National Hip Fracture Database
LOS: Length of Stay
RT-PCR: Real Time Polymerase Chain Reaction
ED: Emergency Department
